# Mixed glycerol and orange peel-based substrate for fed-batch microbial biodiesel production

**DOI:** 10.1016/j.heliyon.2020.e04801

**Published:** 2020-09-14

**Authors:** Eleonora Carota, Maurizio Petruccioli, Alessandro D'Annibale, Silvia Crognale

**Affiliations:** Department for Innovation in Biological, Agro-food and Forest Systems (DIBAF), University of Tuscia, Via S. Camillo De Lellis snc, 01100, Viterbo, Italy

**Keywords:** Biotechnology, Microbiology, Waste treatment, Green engineering, Sustainable development, Microbial biotechnology, Biofuel, Orange peel waste, Biodiesel, Fed-batch process, *Rhodosporidium toruloides*, Oleaginous yeasts, Circular economy

## Abstract

The aqueous extraction of orange peel waste (OPW), the byproduct of the juice extraction process generated annually in massive amounts (21 Mton), yields a carbohydrate-rich liquid fraction, termed orange peel extract (OPE). Several studies highlight that the combination of glycerol, a biodiesel byproduct, with carbohydrate mixtures might boost microbial lipid production. This study performed first a shaken flask screening of 15 oleaginous yeast strains based on their growth and lipid-producing abilities on OPE- and glycerol-based media. This screening enabled the selection of *R. toruloides* NRRL 1091 for the assessment of the process transfer in a stirred tank reactor (STR). This assessment relied, in particular, on either single- and double-stage feeding fed-batch (SSF-FB and DSF-FB, respectively) processes where OPE served as the primary medium and nitrogen-containing glycerol-OPE mixtures as the feeding one. The continuous supply mode at low dilution rates (0.02 and 0.01 h^−1^ for SSF-FB and DSF-FB, respectively) starting from the end of the exponential growth of the initial batch phase enabled the temporal extension of biomass and lipid production. The SSF-FB and DSF-FB processes attained high biomass and lipid volumetric productions (LVP) and ensured significant lipid accumulation on a dry cell basis (Y_L/X_). The SSF-FB process led to LVP of 20.6 g L^−1^ after 104 h with volumetric productivity (*r*_L_) of 0.20 g L^−1^ h^−1^ and Y_L/X_ of 0.80; the DSF-FB process yielded LVP, *r*_L_ and Y_L/X_ values equal to 15.92 g L^−1^, 0.11 g L^−1^ h^−1^ and 0.65, respectively. The fatty acid profiles of lipids from both fed-batch processes were not significantly different and resembled that of *Jatropha* oil, a vastly used feedstock for biodiesel production. These results suggest that OPE constitutes an excellent basis for the fed-batch production of *R. toruloides* lipids, and this process might afford a further option in OPW-based biorefinery.

## 1 Introduction

Second-generation biodiesel, derived from microbial oils, continues to arouse significant interest since it is deemed to be more sustainable than that based on oleaginous plants (Dourou et al., 2018). The use of oleaginous plants for biofuel purposes, in fact, triggers the well-known food vs. fuel conflict that implies the diversion of arable land, irrigation water, and labor to the production of biofuels rather than to food supply. In addition to this, as opposed to oils from plant sources, microbial cultivation is independent of climate conditions, seasonality of production, and geographic location (Atabani et al., 2012). Moreover, the price imbalance caused by the increasing demand for plant oils or animal fat as substitutes for petroleum is a further factor that has aroused interest in the development of microbial-based lipid production. A driving force for the production of a given biofuel is also the opportunity to reduce the emissions of greenhouse gases (GHG), possibly. A comparative calculation of the Net Energy Balances (NEB) and GHG emissions derived from conventional fuels and biofuel technologies showed that the NEB values were higher for microbial biodiesel production than for other biofuels, such as biodiesel from soybean oil and cellulosic ethanol, and that this was associated with a more substantial decrease of GHG emissions (Caspeta and Nielsen, 2013).

Among lipid-accumulating microorganisms, research efforts focused on oleaginous yeasts due to their high growth rates, ability to proliferate on a variety of media, ease of cultivation, and amenability to scale-up (Beopoulos et al., 2011). Besides, the fatty methyl ester profiles of their lipids give excellent biodiesel properties (Karamerou and Webb, 2019).

For these reasons, a wide array of studies focused on the suitability of synthetic cultivation media in supporting growth and oil-producing capability of oleaginous yeasts (Ageitos et al., 2011; Karamerou and Webb, 2019). However, the growth medium can impact on the process economics up to 30% of the total costs. As a consequence, the development of growth media, relying on either cheap or zero-cost feedstocks, is among the current challenges within the frame of microbial oil production (Karamerou and Webb, 2019). Glycerol, a by-product of biodiesel production, represents an appealing feedstock either as the primary carbon source or as a supplement in production media to stimulate lipid accumulation. On the one hand, successful lipid production processes, based on glycerol as the sole carbon source, have been set up for some specific yeast species, such as *Cryptococcus curvatus* (Liang et al., 2010), *Yarrowia lipolytica* (Fontanille et al., 2012), *Rhodosporidium toruloides* (Xu et al., 2012; Yang et al., 2014; Tchakouteu et al., 2015), *Rhodotorula glutinis* (Lorenz et al., 2017) and *Lipomyces starkeyi* (Tchakouteu et al., 2015). On the other hand, in several other oleaginous species, the use of glycerol as the sole carbon source has led to limited biomass production, extended fermentation times and low yields compared to other carbon sources (Chatzifragkou et al., 2011; Lorenz et al., 2017; Papanikolaou and Aggelis, 2011).

Interestingly, several studies found that growth media based on the combination of glycerol with easily assimilable sugars supported both the trophophase and the lipogenic phase. In particular, Galafassi et al. (2012) observed that the supplementation of several sugar-based media with glycerol (5 g L^−1^) in *Rhodotorula graminis* led to increased lipid productivity and yield compared to the non-supplemented media. In another study with *R. toruloides*, the blend of glucose with glycerol led to a 26% increase of lipid yield as compared to that observed in the same medium containing only glucose (Bommareddy et al., 2015).

Although these studies showed the possibility of improving the microbial lipid production processes by concomitant use of glycerol and easily assimilable sugars, their impact is questionable since they generally used glucose. In this respect, the bulk price of glucose ranges from 400 to 800 US$ per ton, thus impacting on the process costs.

Consequently, there is a need to identify low-cost sources of readily assimilable sugars. Orange peel waste (OPW), the solid byproduct of the orange juice extraction process, might represent a potential feedstock for this purpose. The annual production of this waste amounts to around 21 million tons (Balu et al., 2012; Santi et al., 2015) and derives from industrial plants mainly located in the USA, Brazil, Spain, and Italy. Despite its diffusion and availability, only a few microbial processes have relied on OPW-based growth media (Torrado et al., 2011; Santi et al., 2014, 2015). One of the main reasons for the under-exploitation of OPW stems from its content in D-limonene, a reported inhibitory compound to microbes, yeasts in particular (Wilkins et al., 2007). However, D-limonene has a wide range of commercial uses (e.g., flavoring agent, solvent, moistener of water-free cleansers), and, for this reason, several recovery approaches, such as the FMC (Food Machinery Corporation, Lakeland, FL) and the Polycitrus extractor (Fratelli Indelicato, Catania, Italy), are currently available. Recent studies have found that the hot water extraction of OPW yields a liquid fraction containing significant amounts of glucose, fructose, and sucrose termed orange peel extract (OPE) (Park et al., 2014; Santi et al., 2015).

Regardless of both type and cost-efficacy of the carbon sources employed in microbial oil production process, several studies claimed that the fed-batch culture mode enabled the achievement of very promising lipid productions (Li et al., 2007; Zhao et al., 2011; Soccol et al., 2017). Fed-batch processes involving either continuous or sequential feeding of the substrate exhibited higher performance than batch cultivation due to their potential capacity of eliminating substrate-associated growth inhibition and/or achieving high cell density culture (Zhao et al., 2010; Fei et al., 2016).

Thus, the present study aimed at evaluating the concomitant use of glycerol and OPE, derived from a limonene-free OPW, in the formulation of liquid production media for oleaginous yeasts and to test different fed-batch strategies. To this aim, several yeast strains were first screened in shaken flask to investigate their growth and lipid-producing abilities on the two individual substrates. This phase enabled us to perform a comparative assessment of the performance on both OPE and glycerol and to identify the most versatile strains. Among these, one was selected for the transfer of the lipid production process to a mechanically agitated reactor, which was operated in a fed-batch mode, using both a dual- and a single-stage feeding strategy. To the best of our knowledge, this is the first report dealing with the combined use of glycerol and OPE as the possible basis of the lipid production medium for oleaginous yeasts.

## 2 Materials and methods

Figure S1 provides an overall process diagram regarding medium preparation from OPW, fermentation trials and subsequent chemical analyses on biomass and culture supernatants.

### 2.1 Microbial strains and maintenance

The oleaginous yeast strains employed in the present study, listed in alphabetical order, were as follows: *Candida rugosa NRRL Y-95*, *Cryptococcus albidus* (UCD 68-150 and UCD 68-174), *Cryptococcus curvatus* NRRL Y-1511, *Cryptococcus laurentii* UCD 68-201, *Lipomyces starkeii* NRRL 11557, *Rhodosporidium toruloides* (NRRL Y- 1091 and NRRL Y-17902), *Rhodotorula glutinis* (UCD 68-255 and DBVPG 3853) *Rhodotorula minuta* UCD 68-280, *Trichosporon. fermentans* NRRL Y-1492, and *Yarrowia lipolytica* (NRRL YB-423, NRRL Y-1095 NRRL Y-7208). All the strains under study were maintained and routinely sub-cultured every month on potato dextrose agar (PDA) slants.

### 2.2 Growth media

#### 2.2.1 Glycerol

Glycerol-based medium used for screening experiment contained (g L^−1^): technical-grade-glycerol, 20; (NH_4_)_2_SO_4_, 0.5; yeast extract (containing 10% total nitrogen), 0.5; NaH_2_PO_4_, 3.0; KH_2_PO_4_, 0.7. After sterilization in an autoclave, 0.1% of a mineral salt solution 1000× was added, containing per liter: 4.4 g ZnSO_4_, 1.1 g MnCl_2_∙4 H_2_O, 0.315 g CuSO_4_, 1.47 g CaCl_2_, 1 g FeSO_4_, and the pH of the medium was adjusted to 5.5.

#### 2.2.2 OPE

Limonene-free OPW was obtained from the company Agrumigel (Barcellona Pozzo di Gotto, Italy). Its proximate composition, determined as described by Santi et al. (2014) and expressed as percent on a dry weight basis, was as follows: moisture, 6.9 ± 0.5; carbohydrates, 58 ± 2.6; protein, 4.9 ± 0.3; lipids, 5.1 ± 0.4; total phenols, 1.4 ± 0.3; crude fibre, 15.8 ± 2.2 and ash, 7.4 ± 0.2. Only exceptions were lipids and crude fibre which were determined as reported elsewhere (Townshend, 1987). This starting material was added with distilled water (ratio 15:100, w/v), homogenized with an Ultra-Turrax T-18 (IKA Labortechnik, Staufen, Germany) at 3000 rev min^−1^ for 30 s, and then incubated in a water bath at 90 °C for 30 min. After cooling, the liquid suspension was centrifuged (6000 x g, 15 min) and filtrated under vacuum through Whatman n. 41. The pH of the aqueous extract thus obtained, from here onwards referred to as OPE, was 4.7 and its dry weight residue amounted to 27.7 ± 0.5 g L^−1^. The total carbon and nitrogen contents of OPE, determined on lyophilised samples by the Flash EA® 1112 nitrogen and carbon analyzer (Thermo Fisher Scientific, Waltham, MA), were 40.6 ± 0.2% and 0.57 ± 0.0%, respectively. For both screening and reactor experiments, the pH was adjusted to 5.5 with few drops of 0.5 M NaOH, and OPE was supplemented with (NH_4_)_2_SO_4_ (1.0 g L^−1^ medium), thus lowering its C/N ratio from 71 to 30. Sugar composition of OPE was as follows (g L^−1^): glucose, 8.5 ± 0.4; sucrose, 8.5 ± 0.4; fructose, 7.2 ± 0.2; mannose, 1.5 ± 0.1, and pectin, 2.5 ± 0.2. The total phenols, total lipids, soluble protein and citric acid contents of OPE were 0.9 ± 0.0, 0.3 ± 0.0, 0.2 ± 0.0, and 0.3 ± 0.0 g L^−1^, respectively.

### 2.3 Culture conditions

#### 2.3.1 Shaken flask screening (SFS) of oleaginous strains

Biomass and lipid production capacities of the strains under study were comparatively tested on both glycerol- and OPE-based growth medium in shaken culture. To produce inocula, 72-h-old PDA slants were added with sterile physiological solution, vortexed for 15 s, and the resulting suspension added to Erlenmeyer flasks (500 ml), containing 100 ml of growth medium to yield an initial value of absorbance at 600 nm (A_600_) of 0.2. Cultures were then incubated at 30 °C in an orbital shaker at 185 rpm for five days, and sampled daily. All the experiments were performed in triplicate.

#### 2.3.2 Bioreactor experiments

Fed-batch cultures were carried out in a 3-L jacketed bench-top stirred tank reactor (STR) (Applikon, Schiedam, NL), filled initially with 1.2 L of the OPE-based medium. The STR was equipped with a stirrer bearing two six-blade Rushton-type turbines (diameter 4.5 cm and blade width and length, 1.4 cm) and three baffles (width 1.4 cm). The air was introduced inside the reactor through a perforated pipe sparger located under the bottom turbine. The top plate was endowed with several probes, including a PT100 sensor for temperature and dissolved oxygen (DO), and pH sensors (Applikon). Initial standard process conditions were as follows: impeller speed, 600 rpm; aeration rate, 1.5 volume of air per volume of medium per minute (vvm); temperature, 30 °C; initial DO levels, 100% of saturation. When needed, an adaptive control acting on the impeller was activated to maintain DO levels above 30% saturation. Before the inoculation, and, when needed, a Silicon Antifoam 289 (0.5 mL L^−1^) (Sigma Chemical Co., St Louis, MO, USA) was added to the medium. An ADI 1030 (Applikon) adaptive/PID digital controller monitored the time courses of temperature, pH, and dissolved oxygen throughout the process. The pre-inoculum was grown at 30 °C in shaken flasks on Potato Dextrose Broth for 24 h, under orbital shaking (185 rpm), and added to the reactor to yield an initial value of A_600_ equal to 1.0.

The initial batch phase in STR took place on the OPE-based medium with a C/N ratio of 30, and the addition of the feeding started as the culture reached the end of the exponential phase. A peristaltic pump continuously added the feeding at a dilution rate (D), defined as the flow of the feeding per h over the culture volume, which varied as a function of the fed-batch approach. The criterion underlying the establishment of the D values was to supply limited amounts of easily assimilable sugars in the attempt of avoiding catabolite repression phenomena and, thus, fostering the uptake of glycerol.

In the dual-stage feeding (DSF), both feeding solutions were added at a D equal to 0.01 h^−1^. The former was a glycerol-OPE mixture (20:80, v/v) with a C/N ratio of 20, obtained by addition of 25.8 g L^−1^ (NH_4_)_2_SO_4_. The latter had the same composition as the former except for the C/N ratio, which was increased to 80 by (NH_4_)_2_SO_4_ addition (6.4 g L^−1^).

In the single-stage feeding (SSF), instead, the reactor was continuously fed (D = 0.02 h^−1^) with a glycerol-OPE mixture (15:85, v/v) the C/N ratio of which had been adjusted to 20 by addition of 20.0 g L^−1^ of (NH_4_)_2_SO_4_.

Regardless of the fed-batch strategy, the feeding was followed by a final batch phase to enable the consumption of the residual substrate and possibly extend the lipogenic phase. Eq. (1) enabled the calculation of glycerol consumption (G_cons_%) in fed-batch experiments:

(1) $$G_{cons}\%=\left\{1-\left[\frac{Gly_{res}}{\left(\frac{F\cdot \Delta t\cdot G_{in}}{V_{T}}\right)}\right]\right\}\cdot 100$$

where G_in_ and G_res_ are the concentrations of glycerol in the feeding and the growth medium at time t, respectively, F is the flow of the feeding, Δt is the time interval, and V_T_ the total volume of the medium. All the reactor experiments were performed in duplicate.

### 2.4 Determination of yields and rates

The biomass yield (Y_X/S_) and product yields, in terms of lipids or biodiesel and either referred to the amount of substrate consumed (Y_L/S_ and Y_B/S_, respectively) or to biomass ((Y_L/X_, and Y_B/X_, respectively) were calculated as described elsewhere (Carota et al., 2018). The average biomass, lipid, and biodiesel volumetric productivities (*r*_X_ and *r*_L_ and *r*_B_ respectively) were calculated at the time of maximal lipid volumetric production (t_max_) according to Carota et al. (2018). The consumption rate of nitrogen (r_N_) was calculated by Eq. (2):

(2) $${\text{r}}_{\text{N}}=\frac{{\text{N}}_{\text{in}}\cdot {\text{V}}_{\text{in}}+\sum_{\text{i}=1}^{\text{N}}{\text{N}}_{\text{f}}\cdot {\text{V}}_{\text{f}}}{\left({\text{V}}_{\text{in}}+\sum_{\text{i}=1}^{\text{N}}{\text{V}}_{\text{f}}\right)\cdot t_{\max}}$$

Where N_in_ and N_f_ are the concentrations of nitrogen in the initial medium and the feeding and V_in_ and V_f_, the respective volumes, and N the number of feeding phases. The cumulative consumption rate of carbon sources (i.e., glucose, fructose, sucrose, and glycerol) was calculated by the summation of that of each component, as shown in Eq. (3):

(3) $${\text{r}}_{\text{Cs}}=\sum_{i=1}^{m}\left(\frac{Cs_{in}\cdot V_{in}+\sum_{i=1}^{N}Cs_{f}\cdot V_{f}}{(V_{in}+\sum_{i=1}^{N}V_{f})\cdot t_{\max}}\right)$$

Where Cs_in_ and Cs_f_ are the concentrations of each carbon source in the initial medium and the feeding, and V_in_ and V_f_, the respective volumes and m and N the number of carbon sources and feeding phases, respectively.

### 2.5 Analytical methods

#### 2.5.1 Analysis of microbial biomass

Biomass was determined gravimetrically after centrifuging culture samples (3 and 5 mL for shaken flask and reactor cultures) in pre-weighed Falcon tubes (8000 x g, 10 min) and washing three times with distilled water. The pellet thus obtained was freeze-dried for 48 h before gravimetric measurements. Quantification of total lipids was performed on the recovered biomass without any extraction step by the method of Izard and Limberger (2003).

Lyophilised cells underwent direct transesterification to yield fatty acid methyl esters (FAME) (Schutter and Dick, 2000) that were then analysed by a Master GC gas chromatograph (DANI Instrument SpA, Cologno Monzese, Italy) equipped with a Rxi-5MS (Restek, Germany) capillary column (0.25 mm id × 30 m length). The temperature program used to separate FAME was as follows: initially isothermal at 89 °C for 2 min, temperature gradient from 89 to 280 °C at a 6 °*C min*^−1^ and, finally isothermal at 280 °C for 5 min. The temperatures of the injector and flame ionization detector were set at 280 and 300 °C, respectively. Each FAME was identified by comparing its retention time with that of commercial standards contained in the FAME Mix C8-C24 (Sigma Aldrich, 18918-1AMP, USA). For quantification purposes, each sample underwent the addition of an internal standard (i.e., methyl nonadecanoate) before the transesterification.

#### 2.5.2 Analysis of the culture supernatants

Culture supernatants were recovered by centrifugation, as described above. Residual contents of total sugars were determined by using the phenol-sulphuric acid method (Dubois et al., 1956). The residual nitrogen content was determined by a modified Kjeldahl method (Domini et al., 2009) involving microwave digestion (MarsXpress, CEM, Matthews, NC, USA) with a mixture of 37% HCl (Carlo Erba Reagenti, Milan, Italy) and 30% H_2_O_2_ (Merck KGaA, Darmstadt, Germany) and the subsequent spectrophotometric determination of ammonium using the nitroprusside method (Anderson and Ingram, 1993). In reactor experiments, sugar consumption was evaluated both by phenol-sulphuric acid assay (Dubois et al., 1956) and using specific enzymatic kits. In particular, the SUCROSE, D-FRUCTOSE and D-GLUCOSE kit (Megazyme International Ireland Ltd, Wicklow, Ireland), was used to determine the volumetric concentration of these sugars in growth media; the Mannose/Sucrose/D-Glucose Assay Kit (Megazyme International Ireland Ltd, Wicklow, Ireland) was used for mannose. Glycerol was determined separately by the K-GCROLGK kit (Megazyme International Ireland Ltd, Wicklow, Ireland). Pectin determination relied on the colorimetric method of Taylor and Buchanan-Smith (1992) calibrated in the 10-200 μg ml^−1^ range.

### 2.6 Empirical equations for the calculation of saponification, iodine value and cetane number

To evaluate the suitability of lipids produced for biodiesel, the empirical Eqs. (4) and (5) for the calculation of saponification (SV) and iodine values (IV), respectively, developed by (Kalayasiri et al., 1996), were used. The estimations of cetane number (CN) and higher heating value (HHV) relied on the empirical Eqs. (6) and (7) developed by Krisnangkura (1986) and Demirbaş (1998), respectively.

(4) $$SN=100\cdot \sum_{i=1}^{N}\left(\frac{560\cdot A_{i}}{MW_{i}}\right)$$

(5) $$IV=100\cdot \sum_{i=1}^{N}\left(\frac{254\cdot D_{i}\cdot A_{i}}{MW_{i}}\right)$$

(6) $$CN=46.3+\frac{5458}{SN}-\left(0.225\cdot IV\right)$$

(7) HHV=49.43−[(0.041⋅SV)+(0.015⋅IV)]

where A_i_, D_i_, and MW_i_ represent the relative percent abundance, the number of double bonds, and the molecular mass of each FAME component, respectively.

## 3 Results

### 3.1 Screening of oleaginous on glycerol- and OPE-based media

Table 1 summarizes the results obtained by growing the 15 putatively oleaginous strains on the glycerol- and OPE-based media. In both cases, the lipid production process was relatively rapid, reaching the product peak within 72 and 96 h from the inoculation, depending on the strain.

**Table 1:** Biomass and lipid production rates (*r*_x_, *r*_L_, respectively), percent lipid contents, referred to dry biomass, percentage of substrate consumed calculated at time of maximal lipid accumulation (t_max_), and yield of product referred to the substrate consumed (Y_L/S_) obtained for each of the 15 yeast strains grown on glycerol- (Gly) or OPE-based medium. The experiments were performed in triplicate, and data between round parenthesis represent the standard deviation of data.

Yeasts	*r*_X_ (g L^−1^ h^−1^⋅10^−3^)		*r*_L_† (g L^−1^ h^−1^⋅10^−3^)		Lipid content † %)		Substrate consumed (%)		Y_L/S_		t_max_ (h)	
	Gly	OPE	Gly	OPE	Gly	OPE	Gly	OPE	Gly	OPE	Gly	OPE
*C. rugosa* NRRL Y-95	52.6Bb (2.1)	64.1FGa (1.5)	4.9Eb (0.0)	22.6FGa (1.3)	9.3FGb (0.4)	35.2FGa (1.2)	34.2Cb (1.0)	72.7Aa (9.2)	0.04Fb (0.00)	0.08EFa (0.01)	72	72
*C. albidus* UCD 68-150	7.3Eb (2.7)	84.2Da (2.6)	1.2Fb (0.1)	21.0Ga (1.1)	16.5EFb (5.2)	24.9Ia (2.1)	26.6Db (1.6)	86.5Aa (10.6)	0.01Gb (0.00)	0.06Fa (0.01)	72	72
*C. albidus* UCD 68-174	12.7Db (0.8)	175.5Aa (3.2)	1.9Fb (0.1)	53.2Ca (4.6)	14.5Eb (1.8)	30.3Ha (3.19)	27.8Db (0.5)	97.4Aa (10.1)	0.02Gb (0.00)	0.14Ca (0.01)	72	72
*C. curvatus* NRRL Y-1511	20.3Cb (2.6)	72.0EFa (3.0)	12.1Db (0.1)	18.1Ga (1.2)	59.8Aa (7.2)	24.5Ib (2.5)	27.1Db (0.5)	93.5Aa (12.3)	0.13Da (0.01)	0.07Fb (0.01)	72	96
*C. laurentii* UCD 68-201	9.4Eb (1.9)	134.4Ba (5.2)	1.7Fb (0.3)	62.7Ba (0.3)	18.6Eb (0.6)	24.2Ia (0.8)	28.2Db (0.6)	89.8Aa (9.7)	0.02Gb (0.00)	0.18Ba (0.01)	72	72
*L. starkeii* NRRL 11557	9.7Eb (3.1)	58.7Ga (9.1)	4.4Eb (0.9)	26.5EFa (2.8)	45.8Ba (5.8)	45.2CDa (2.12)	28.8Db (1.2)	80.7Aa (8.8)	0.06Eb (0.00)	0.11DEa (0.01)	96	72
*R. torouloides* NRRL Y-1091	23.9Cb (0.3)	103.3Ca (0.5)	15.4Cb (0.5)	79.7Aa (0.6)	64.2Ab (2.9)	77.1Aa (1.0)	28.3Db (0.6)	91.7Aa (9.9)	0.16Cb (0.01)	0.23Aa (0.02)	72	72
*R. torouloides* NRRL Y-17902	12.2Db (1.1)	65.0FGa (2.9)	2.7Fb (0.3)	28.4EFa (1.6)	24.3DEb (0.5)	43.6DEa (0.5)	28.4Db (0.8)	85.1Aa (8.8)	0.03bF (0.00)	0.09DEa (0.01)	72	72
*R. glutinis* DBVPG 3853	19.1Cb (0.9)	95.1Ca (2.1)	6.5Eb (0.8)	46.2Da (5.3)	33.7CDb (2.4)	48.6Ca (6.7)	27.0Db (0.6)	88.3Aa (9.7)	0.07Eb (0.01)	0.14Ca (0.02)	72	72
*R. glutinis* UCD 68-255	45.4Ba (2.2)	44.6Ha (0.1)	4.9Eb (0.4)	18.5Ga (0.2)	10.8Fa (1.4)	11.3Ma (0.1)	47.9Ab (0.9)	90.0Aa (9.7)	0.03Fb (0.00)	0.05Fa (0.00)	72	72
*R. minuta* UCD 68-280	20.9Cb (2.7)	139.4Ba (8.4)	6.0Eb (0.5)	43.8Da (5.1)	28.9Ca (6.0)	31.4GHa (1.7)	28.8Db (0.8)	84.1Aa (9.2)	0.06Eb (0.00)	0.13CDa (0.01)	72	96
*T. fermentans* NRRL Y-1492	90.3Aa (6.9)	99.7Ca (6.7)	6.0Eb (1.2)	16.5Ga (1.2)	6.6Gb (0.8)	16.6La (0.1)	39.7Bb (0.6)	82.3Aa (8.8)	0.04Fa (0.00)	0.05Fa (0.01)	72	72
*Y. lipolytica* NRRL Y-1095	54.0Bb (4.2)	74.7Ea (0.7)	19.2Bb (1.1)	29.8Ea (2.0)	35.6Ca (4.0)	39.9EFa (2.2)	28.8Db (0.9)	77.5Aa (8.1)	0.19Ba (0.01)	0.10EFb (0.01)	72	72
*Y. lipolytica* NRRL Y-7208	17.3Cb (2.7)	85.5Da (3.0)	7.0bE (1.2)	23.2FGa (2.8)	40.6BCa (0.3)	27.1HIb (2.3)	27.4Db (0.6)	75.2Aa (9.4)	0.07Ea (0.01)	0.08EFa (0.02)	72	72
*Y. lipolytica* NRRL YB-423	53.0Ba (9.0)	44.6Ha (1.0)	33.6Aa (3.2)	24.5Fb (1.0)	63.4Aa (4.8)	55.0Bb (1.0)	28.8Db (1.2)	89.7Aa (9.6)	0.34Ab (0.02)	0.07Fa (0.00)	72	72

Same uppercase letters indicate lack of statistically significant differences among column means; same lowercase letters indicate lack of statistically significant differences between values of the same parameter obtained on the OPE- or glycerol-based medium.

† *r*_L_ and percent lipid content referred to cell dry weight were calculated from the values of volumetric lipid amount obtained by the method of Izard and Limberger (2003). Multiple pair-wise comparisons were performed by the Tukey test (P ≤ 0.05).

The majority of strains exhibited a limited ability to use glycerol as the carbon source. The extent of glycerol consumed at the product peak did not reach one-third of its original content in the medium except for *R. glutinis* UCD 68-255 and *T. fermentans* cultures where glycerol consumption amounted to 47.9 and 39.7%, respectively. In the latter strain, the significant consumption of the carbon source was reflected in the highest biomass production rate while the former gave similar results to those found for *C. rugosa*, *P. anomala*, *R glutinis* UCD-68255, and two *Y. lipolytica* strains (i.e., Y-1095 and YB-423). Among the 15 strains, only 9 met the requirement of oleaginicity on the glycerol-based medium, and the highest lipid contents referred to dry biomass were found in *R. toruloides* NRRL 1091, *Y. lipolytica* YB-423 and *C. curvatus* (64.2, 63.4, and 59.8%, respectively) (Table 1). Among the tested strains, *Y. lipolytica* YB-423 that stood out for its lipid production rate also led to Y_L/S_ values of 0.34 ± 0.02 g g^−1^, which were close to the maximum conversion yield achievable on glycerol (Bommareddy et al., 2015). However, also *Y. lipolytica* NRRL Y-1095, *R. toruloides* NRRL Y-1091, and *C. curvatus* NRRL Y-1511, demonstrated a satisfactory capacity to convert glycerol to storage lipids, with Y_L/S_ of 0.19, 0.16 and 0.13 g g^−1^, respectively (data not shown). Noteworthy, *T. fermentans* that had exhibited the highest biomass production rate had a lipid content as low as 6.6%.

As opposed to the glycerol-based medium, on the OPE-based medium, the carbon sources, mainly represented by fructose, glucose, and sucrose, were significantly depleted from the medium with percent consumptions ranging from around 73 to 97%. Table 1 also shows that the OPE-based medium enabled comparatively higher biomass productivity than that observed on the glycerol-based one. The strains that exhibited the highest biomass productivities in the OPE-based medium were *C. albidus*, *R. minuta*, and *C. laurentii*, with *r*_X_ values being 0.18, 0.14, and 0.13 g L^−1^ h^−1^, respectively. Except for *R. glutinis* UCD 68-255 and *T. fermentans*, all the strains tested met the requirement of oleaginicity in the OPE-based medium. The highest lipid contents were found in *R. toruloides* NRRL 1091 (77%) and *Y. lipolytica* NRRL YB-423 (55%); the former strain also exhibited the highest lipid production rate (0.08 g L^−1^ h^−1^) and an Y_L/S_ equal to 0.31 g g^−1^.

*R. toruloides* NRRL 1091 exhibited the best performance on the OPE-based medium and, at the same time, had a high lipid-accumulating capacity on both media. Thus, due to its versatility, this strain was selected for the transfer of the lipid production process in a stirred tank reactor operated in the fed-batch mode.

### 3.2 Process transfer to the reactor operated in fed-batch mode

Shaken flask experiments with *R. toruloides* NRRL 1091 had shown a high-speed consumption of the carbon sources in the OPE-based medium associated with promising yields of lipid production. Thus, the subsequent transfer to the reactor aimed to increase the biomass density and extend the lipid accumulation phase. These objectives relied on fed-batch strategies supplying the carbon and nitrogen sources in a controlled way.

#### 3.2.1 Fed-batch with double stage feeding (DSF-FB)

**Figure 1:**
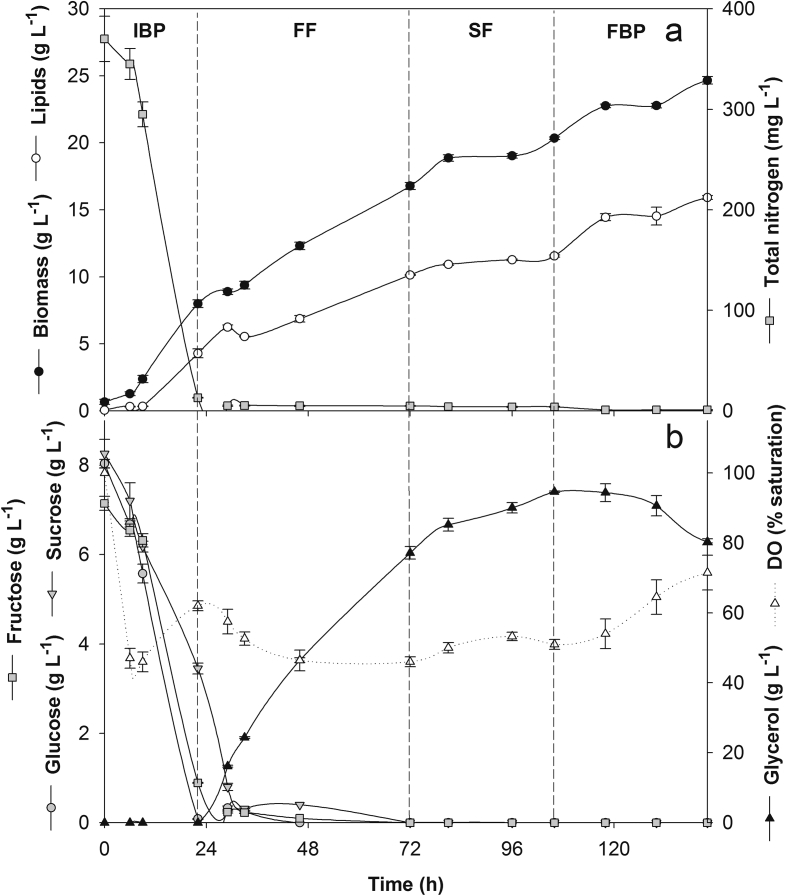
(a) Time courses of lipid and biomass production and nitrogen consumption by *R. toruloides* NRRL 1091 grown in a stirred tank reactor operated in the double-stage fed-batch mode; (b) time courses of glucose, sucrose, fructose, and glycerol consumptions and dissolved oxygen (DO) levels. Vertical dashed lines separate the following phases of the process: initial batch phase (IBP), first and second feeding (SF and FF, respectively) and the final batch phase (FBP). Data are the means of duplicate reactor experiments and error bars express the respective standard deviations.

The primary medium for the initial batch phase (IBP) was the OPE-based medium with C/N 30. Although both feeding media, supplied to *R. toruloides* cultures at a D of 0.01 h^−1^, relied on the same OPE-glycerol mixture (80:20, v/v), they differed for their C/N ratio which was 20 in the first and 80 in the second feeding. At the end of the IBP, biomass production amounted to 8.0 ± 0.9 g L^−1^, and an almost quantitatively depletion of both glucose and fructose took place, while residual sucrose amounted to 64.5% of its original content (Figure 1a). The fast growth observed in IBP also led to a rapid depletion of nitrogen, and, at the end of it, volumetric lipid production, r_L_, and Y_P/X_ amounted to 4.29 g L^−1^, 0.20 g L^−1^ h^−1^, and 53.7 ± 2%. The first feeding started at the end of the exponential growth of the IBP, when the profile of DO inverted its trend and started to increase (Figure 1b), in the attempt to extend its duration and attain a higher cell density. This expectation was met since biomass production continued to increase throughout the whole first feeding phase, albeit at a slightly lower rate than that of IBP. At the end of this phase (72 h from the inoculation), biomass and lipid productions amounted to 16.8 ± 0.3 and 10.14 g L^−1^, respectively (Figure 1a); their respective productivities, however, were lower than those at the end of the IBP (0.23 vs. 0.33 g L^−1^ h^−1^ and 0.15 vs. 0.20 g L^−1^ h^−1^) while the Y_L/X_ (0.60 vs. 0.54) significantly differed (*P* = 0.026). The low D adopted enabled high-speed consumption of all the sugars from the OPE component of the feeding so that their residual concentrations in the medium were undetectable (Figure 1b). Conversely, although the glycerol content in the medium raised significantly, its consumption rate was higher than that observed in the shaken flask (0.17 vs. 0.08 g L^−1^ h^−1^). At the end of the second feeding, despite a high-speed consumption of OPE sugars and increased consumption rate of glycerol (0.21 g L^−1^ h^−1^), both r_L_ and Y_L/X_ (0.08 g L^−1^ h^−1^ and 0.57, respectively) decreased as compared to those of the first feeding. Likely, the abrupt increase in the C/N value of the medium in this phase (from 541 at 72 h to 2208 at 104 h) exerted a negative impact on lipid accumulation. The second feeding was followed by a final batch phase, lasting 60 h, aimed to exploit the residual nutrients derived from the end of the second feeding. During this phase, the glycerol consumption rate was 0.40 g L^−1^ h^−1^, and the volumetric lipid production reached its peak (15.92 g L^−1^ at 142 h) in concomitance with a biomass production of 24.65 g L^−1^ (Figure 1a), and Y_L/X_ of 0.65 (Table2). To assess whether the properties of the product were affected throughout the process, the FAME profiles were determined at the end of each culture phase; Table 2 shows that the increase in C18:0 and 18:1 and a concomitant decrease of 16:0 and 18:2 observed over the different culture phases, did not exert a significant impact on the estimated biodiesel parameters.

**Table 2:** Specific product yield (Y_L/X_), fatty acid profiles and biodiesel parameters thereof derived as a function of cultures phases in a double stage fed-batch process conducted with *R. toruloides* in a 3-L stirred tank rector. Abbreviations of biodiesel parameters and related expression units are as follows: saponification value (SV, mg KOH g^−1^ oil), iodine value (IV, g I_2_ (100 g)^−1^ cetane number (CN) and higher heating value (HHV, MJ kg^−1^).

Culture Phase	Culture time (h)	Y_L/X_‡	Percent abundance of FAME in lipid profiles						Biodiesel parameters			
			C16:1†	C16:0†	C18:2†	C18:1†	C18:0†	Other SFA†§	SV	IV	CN	HHV
Batch	0–22	0.54^a^ (0.04)	0.63^a^ (0.03)	32.18^b^ (0.23)	13.50^b^ (0.07)	43.98^a^ (0.10)	6.06^a^ (0.26)	3.65^c^ (0.17)	206.24	64.63	58.22	41.94
First feeding	23–72	0.60^b^ (0.02)	0.58^a^ (0.09)	26.20^a^ (2.74)	10.29^a^ (0.37)	50.95^b^ (2.02)	9.04^b^ (1.00)	2.93^b^ (0.56)	204.89	65.04	58.31	42.00
Second feeding	73–106	0.57^ab^ (0.0)	0.65^a^ (0.04)	27.40^a^ (2.38)	9.83^a^ (0.04)	50.58^b^ (0.16)	9.03^b^ (0.30)	2.51^b^ (0.04)	204.94	63.94	58.55	41.99
Nutrient depletion phase	106–142	0.65^b^ (0.02)	0.66^a^ (0.08)	24.28^a^ (1.34)	8.84^a^ (2.99)	54.67^c^ (1.22)	9.73^b^ (0.28)	1.78^a^ (0.20)	204.31	65.83	58.20	42.04

Same superscript letters indicate lack of statistically significant differences among column means. SV, IV, CN and HHV were estimated using the models developed by Kalayasiri et al. (1996), Krisnangkura (1986) and Demirbas (1998), respectively.

† Data are the means of 6 chromatographic runs (3 technical replicates for each reactor experiment) and respective standard deviations are shown between round brackets.

§ SFA, saturated fatty acids.

‡ Y_L/X_ was calculated based on the biomass and lipid volumetric concentration obtained at the end of each phase. Lipid concentration was determined by the method of Izard and Limberger (2003). Multiple pair-wise comparisons of specific product yield and percentage concentrations of each fatty acid along time were performed by the Tukey test (P ≤ 0.05).

#### 3.2.2 Fed-batch with single stage feeding (SSF-FB)

**Figure 2:**
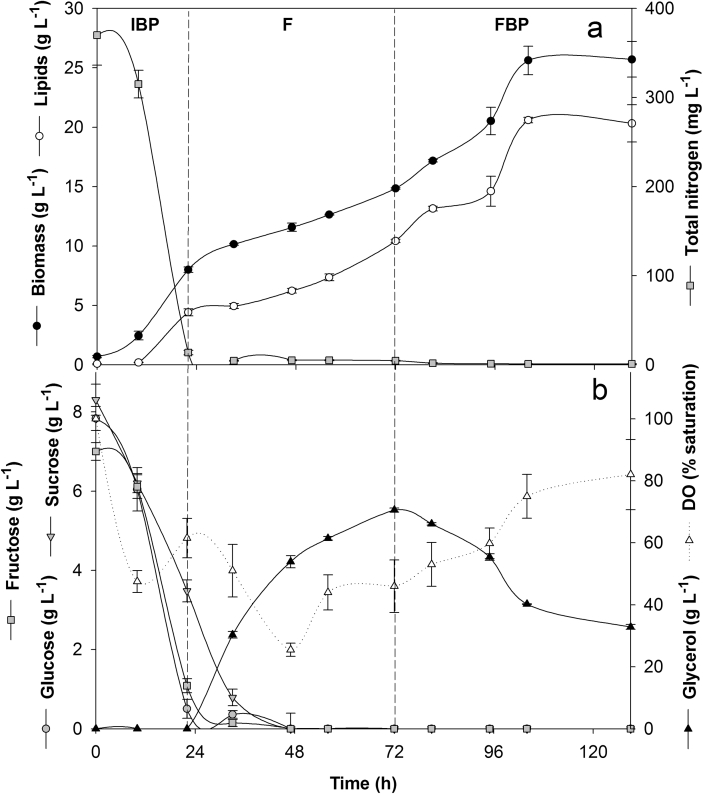
(a) Time courses of lipid and biomass production and nitrogen consumption by *R. toruloides* NRRL 1091 grown in a stirred tank reactor operated in the single-stage fed-batch mode; (b) time courses of glucose, sucrose, fructose, and glycerol consumptions and dissolved oxygen (DO) levels. Vertical dashed lines separate the following phases of the process: initial batch phase (IBP), feeding (F) and the final batch phase (FBP). Data are the means of duplicate reactor experiments and error bars express the respective standard deviations.

In SSF-FB experiments, the relative proportion of glycerol in the feeding solution was reduced to yield an 85:15 (v/v) OPE–glycerol mixture with a C/N ratio of 20. This adjustment derived from the observation that extents of glycerol supplied were in excess compared to those consumed. This feeding solution was added to the reactor with a D value twice as high that of DSF-FB since the increased supply rate of easily assimilable sugars was expected to impact positively on biomass production rate. Biomass and lipid productivities and carbohydrate consumption profiles at the end of the IBP (0–22 h) were similar to those obtained in the DSF-FB process, as expected (Figures 2a and 2b). Also in this case, the feeding promoted a further increase in both biomass and lipid productions (Figure 2a), reaching values as high as 14.84 ± 0.06 and 10.43 ± 0.15 g L^−1^. The expectation of faster growth kinetics as a consequence of the increased D value was not met since the r_X_ value did not differ from than that observed in the first feeding of the DSF-FB (0.21 vs. 0.23 g L^−1^ h^−1^) (*P* = 0.184); likewise, r_L_ values were similar (0.16 vs. 0.15 g L^−1^ h^−1^). Despite the increased dilution rate, sugars from the OPE component were rapidly consumed downstream of their introduction into the system (Figure 2b), and the extent of glycerol consumption increased up to 0.25 g L^−1^ h^−1^. As already done for DSF-FB, the duration of the process was extended beyond the end of the feeding. At the end of this final batch phase, the biomass productivity increased again to yield values close to those observed in the IBP (0.27 vs. 0.33 g L^−1^ h^−1^), while the lipid productivity increased to 0.28 g L^−1^ h^−1^, reaching the highest value of the whole fermentation. During this phase, the glycerol consumption rate (0.95 g L^−1^ h^−1^) was markedly higher than that of the feeding. Biomass and lipids peaked after 104 h, and their respective volumetric productions were 25.62 ± 1.2 and 20.6 ± 0.23 g L^−1^ while Y_L/X_ amounted to 0.80 (Figure 2a). Table 3 shows that even though some temporal changes in the FAME profiles took place, they did not negatively impact the estimated biodiesel properties, the values of which fulfilled the Standard EN14214.

**Table 3:** Specific product yield (Y_L/X_), fatty acid profiles and biodiesel parameters thereof derived as a function of cultures phases in a single stage fed-batch process conducted with *R. toruloides* in a 3-L stirred tank rector. Abbreviations of biodiesel parameters and related expression units are as follows: saponification value (SV, mg KOH g^−1^ oil), iodine value (IV, g I_2_ (100 g)^−1^ cetane number (CN) and higher heating value (HHV, MJ kg^−1^).

Culture Phase	Culture time (h)	Y_L/X_‡	Percent abundance of FAME in lipid profiles						Biodiesel parameters			
			C16:1†	C16:0†	C18:2†	C18:1†	C18:0†	Other SFA†§	SV	IV	CN	HHV
Batch	0–22	0.55^a^ (0.05)	0.36^a^ (0.07)	27.14^b^ (0.55)	13.82^c^ (0.25)	49.29^a^ (0.43)	7.00^a^ (0.25)	2.52^c^ (0.20)	204.72	69.72	57.27	42.08
First feeding	23–72	0.70^b^ (0.02)	0.53^b^ (0.06)	24.17^a^ (0.46)	9.74^a^ (0.74)	55.15^b^ (0.69)	8.27^b^ (0.40)	2.15^b^ (0.08)	203.67	67.76	57.85	42.10
Final batch phase	73–104	0.80^c^ (0.02)	0.52^b^ (0.02)	23.45^a^ (0.06)	10.11^b^ (0.70)	54.99^b^ (0.92)	8.82^b^ (0.25)	2.11^a^ (0.05)	203.44	68.28	57.76	42.11

Same superscript letters indicate lack of statistically significant difference. SV, IV, CN and HHV were estimated using the models developed by Kalayasiri et al. (1996), Krisnangkura (1986) and Demirbaş (1998), respectively.

† Data are the means of 6 chromatographic runs (3 technical replicates for each reactor experiment) and respective standard deviations are shown between round brackets.

§ SFA, saturated fatty acids.

‡ Y_L/X_ was calculated based on the biomass and lipid volumetric concentration obtained at the end of each phase. Lipid concentration was estimated by Izard and Limberger (2003) method. Multiple pair-wise comparisons of specific product yields and percentage concentrations of each fatty acid along time were performed by the Tukey test (P ≤ 0.05).

Table 4 reports the comparison between performance indicators of biodiesel production of the two fed-batch strategies calculated at the respective lipid peaks. The product yields, in particular, refer to biodiesel. Noteworthy, the biodiesel yields of the lipids obtained in the DSF-FB and SSF-FB were very high (93.5 and 88%, respectively) and, above all, their Y_L/X_ were 0.65 and 0.80, respectively. The FAME profiles obtained at the lipid peak in the DSF-FB and SSF-FB processes did not significantly differ (Table 4).

**Table 4:** Performance indicators of biodiesel production process in STR by *R. toruloides* in fed-batch experiments, operated either in double or single-stage feeding (DSF and SSF, respectively) including yields (Biodiesel yield, Y_L/S_, Y_L/X_, Y_X/S_) and biodiesel and biomass production rates (r_L_ and r_X_, respectively), nitrogen and total carbon sources consumption rates (r_N_, r_S_), and percent fatty acid composition. All values have been calculated at the time of maximal lipid production.

Parameter	Dimension unit	DSF values	SSF values
Yields			
Biodiesel yield	(%)	93.54 ± 1.48^b^	88.00 ± 2.68^a^
Y_L/X_ǂ	(g g^−1^)	0.65 ± 0.01^a^	0.80 ± 0.00^b^
Y_L/S_ǂ	(g g^−1^)	0.29 ± 0.00^a^	0.29 ± 0.00^a^
Y_X/S_ǂ	(g g^−1^)	0.45 ± 0.00^b^	0.37 ± 0.00^a^
Rates§			
r_L_ǂ	g L^−1^ h^−1^	0.11 ± 0.00^a^	0.20 ± 0.00^b^
r_S_ǂ	g L^−1^ h^−1^	0.38 ± 0.00^a^	0.50 ± 0.00^b^
r_N_ǂ	mg L^−1^ h^−1^	14.13 ± 2.20^a^	19.94 ± 2.10^b^
r_X_ǂ	g L^−1^ h^−1^	0.17 ± 0.00^a^	0.24 ± 0.00^b^
Lipid profile†§			
Palmitoleic acid	(%)	0.66 ± 0.08^a^	0.52 ± 0.02^a^
Palmitic acid	(%)	24.28 ± 1.34^a^	23.45 ± 0.06^a^
Linoleic acid	(%)	8.84 ± 2.99^a^	10.11 ± 0.70^a^
Oleic acid	(%)	54.67 ± 1.22^a^	54.99 ± 0.92^a^
Stearic acid	(%)	9.73 ± 0.28^b^	8.82 ± 0.25^a^
Other SFA	(%)	1.93 ± 0.30^a^	2.11 ± 0.05^a^
Other MUFA	(%)	n.d.	n.d.
Other PUFA	(%)	n.d.	n.d.

Pair-wise comparisons among yields, rates and single fatty acids were performed by the Tukey test (P ≤ 0.05). Same superscript letters indicate lack of statistically significant difference within row means; n.d., not detected.

ǂ Data are the means ± SD of duplicate reactor experiments.

† Data are the means ± SD of 6 determinations (3 technical replicates for each reactor experiment.

§ Predominant fatty acids are listed as a function of increasing retention time.

## 4 Discussion

The two byproducts under scrutiny in this investigation have different profiles concerning their exploitation for the second- and third-generation biodiesel production. On the one hand, OPE has been used for this purpose by two studies, one of which conducted with filamentous fungi (Carota et al., 2018) and the other one with algae (Park et al., 2014). On the other hand, instead, glycerol is probably one of the most exploited growth substrates for microbial lipid production due to its lower costs than other carbon sources and ease of preparation of glycerol-based media. Furthermore, it is the main byproduct of biodiesel manufacturing and, thus, its recycling contributes to lower production costs and is compliant with the circular economy concept (Leiva-Candia et al., 2014). A variety of lipid production studies used either pure (Patel et al., 2017) or crude glycerol (André et al., 2009; Chatzifragkou et al., 2011) and others focused on the comparison of process performance with the two formulations (Signori et al., 2016; Xu et al., 2012). Taking into account the extreme variability in the composition of the crude glycerol, as far as impurities are concerned (Xu et al., 2012), the present study used technical-grade glycerol deliberately as a trade-off between the two options.

The screening phase had a dual purpose. On the one hand, to select one of the two matrices as the main component of the medium intended for the initial batch phase of the subsequent fed-batch reactor processes. On the other hand, to select a strain based on its growth and lipid-producing versatility on both media, taking into account that the feeds consisted of a mixture of the two matrices. Concerning the initial medium for fed-batch processes, several studies included yeast extract (YE) or peptone as nitrogen sources to promote fast growth kinetics (Li et al., 2007; Zhao et al., 2011; Galafassi et al., 2012; Dias et al., 2015; Fei et al., 2016). Although peptone and YE are excellent sources of complex nutrients for fast growth at the lab-scale, their cost may be limiting for lipid production on an industrial scale, and thus this study selected ammonium sulphate to adjust the C/N ratios of the two media. In fact, besides to its positive impact on both biomass growth and lipid accumulation in several yeast species (Kitcha and Cheirsilp, 2011; Papanikolaou and Aggelis, 2002), ammonium sulphate is one of the cheapest nitrogen sources and thus its use has a limited impact on process costs (Leiva-Candia et al., 2014).

The shaken flask screening conducted here with several oleaginous yeasts on the two byproducts separately showed that OPE was generally more conducive than glycerol to both growth and lipid production. First of all, the vast majority of the strains under study fulfilled the oleaginicity criterion on the OPE-based medium, while only ten out of fifteen did the same on glycerol. With only exceptions of the *Y. lipolytica* strains and *C. curvatus*, Y_L/X_ values on OPE were higher than or equal to those on glycerol. Among the strains under study, *R. toruloides* NRRL 1091 performed best on the OPE-based medium and maintained a high lipid-accumulating capacity on glycerol. For these reasons, we selected this strain for the transfer of the lipid production process in an STR operated in the fed-batch mode where OPE acted as the initial medium, and the subsequent feeding/s relied on a glycerol-OPE mixture. A variety of studies claim a high capacity of this species of growing and accumulating lipids on a range of carbon sources, including hexoses (glucose, fructose, and galactose) (Li et al., 2007; Bommareddy et al., 2015), pentoses (xylose) (Wiebe et al., 2012) and polyols such as glycerol (Yang et al., 2014). Moreover, several *R. toruloides* strains maintained high lipid-producing performance when grown on byproduct-derived liquid production media, such as sugarcane juice (Soccol et al., 2017), molasses (Vieira et al., 2014), sugarcane bagasse hemicellulosic hydrolysate (Bonturi et al., 2017) and, last but not least, on glycerol derived from biodiesel manufacturing (Xu et al., 2012; Yang et al., 2014).

In the present study, the rationale underlying the setup of the fed-batch processes was to use the OPE-based medium for the initial batch phase to enable rapid exponential growth. In this respect, *R. toruloides* NRRL-1091 performed high-speed and simultaneous consumption of all the sugars in OPE in accordance to some investigations performed with other strains of the same species on growth media made of mixtures of hexoses and disaccharides (Matsakas et al., 2015; Tsakona et al., 2019). In particular, *R. toruloides* DSM 4444 grown on raw hydrolyzates derived from confectionery wastes, concurrently consumed glucose, fructose, and galactose without catabolite repression (Tsakona et al., 2019).

A further assumption of this study relied on the evidence provided by some studies that glycerol supplementation to a sugar-based medium might promote lipid accumulation in oleaginous yeasts (Galafassi et al., 2012; Rakicka et al., 2015). As a consequence, the present study used nitrogen-supplemented glycerol-OPE mixtures as the feedings to promote both the achievement of high-cell density cultures and prolongation of the lipid accumulation phase. The mixed substrate cultivation has the dual advantage of associating efficient cell mass production, generally maximized with glucose, with high lipid content, promoted by glycerol, as shown in *R. toruloides* DSMZ4444 cultures (Bommareddy et al., 2015).

In both fed-batch processes, the reactor was continuously fed with the feeding solutions at low dilution rates to allow a rapid consumption of OPE sugars to avoid catabolite repression phenomena, already reported in *R. toruloides* (Bommareddy et al., 2015). Regardless of the fed-batch process, this study used nitrogen-containing feedings; the adopted dilution rates, however, enabled the maintenance of nitrogen-limiting conditions in the medium, thus allowing a temporal extension of the exponential growth without impairing the accumulation of lipids. Wiebe et al. (2012) observed that *R. toruloides* CBS14 fed-batch cultures grown on a glucose-based medium at C/N 80 and subjected to a constant and low nitrogen supply led to the highest total lipid production and best substrate consumption. Analogous to the study of Wiebe et al. (2012), the present investigation relied on the addition of ammonium sulphate to adjust the C/N ratios. Although the dilution rate avoided the accumulation of OPE sugars in the medium, glycerol utilization was low during the feeding phases of both fed-batch processes, probably due to the phenomenon of catabolite repression mentioned above. The rapid increase in glycerol consumption rate immediately downstream of the end of the feeding, namely in the final batch phase, suggests that this mechanism was operational in our fed-batch experiments.

Noteworthy, the lipid product peak occurred during the final batch phase in both fed-batch processes when glycerol consumption was significantly higher than in the previous culture phases, leading to r_L_ values as high as 0.20 and 0.11 g L^−1^ h^−1^ in SSF-FB and DSF-FB, respectively. In this respect, our results resemble those obtained by Signori et al. (2016), who also observed the attainment of lipid peak after the cessation of the feeding phase in processes conducted with both crude and pure glycerol. The lipid productivity of the SSF-FB was similar to that reported for the CBS14 strain grown on a glucose-based medium and supplied with a feeding solution at C/N 80 (Wiebe et al., 2012) and higher than a glycerol-based fed-batch process with *R. toruloides* DSM4444 (Leiva-Candia et al., 2015); however, it was lower than those attained in *R. toruloides* Y4 fed-batch cultures on glucose-based media that yielded *r*_L_ values as high as ~0.5 g L^−1^ h^−1^ (Li et al., 2007; Zhao et al., 2011). At least two reasons might provide a plausible explanation of the superior performance of the Y4 strain, apart from strain-related differences. First of all, both Li et al. (2007) and Zhao et al. (2010) used glucose-based media containing significant amounts of peptone and/or YE for the initial batch phase; moreover, the concentrations of the carbon source were higher both in the initial medium and in the feedings than those of the present study. The latter reason might also explain the lower productivities of the fed-batch processes of this study than those with other *R. toruloides* strains grown on raw carbohydrate mixtures, such as Jerusalem artichoke extracts (Zhao et al., 2010), lignocellulose hydrolysates from corn stover (Fei et al., 2016), and sugarcane juice (Soccol et al., 2017). However, the SSF-FB enabled high lipid accumulation in *R. toruloides* NRRL 1091 cells, which were similar to those found in other strains of the same species grown on media either based on glucose (Li et al., 2006; Wiebe et al., 2012) or fatty acid and corn steep liquor (Picataggio and Smittle, 1979). Moreover, the performance of *R. toruloides* NRRL 1091 operated in the SSF-FB mode was superior over that attained by batch cultures of the same strain grown in an STR on an OPE-based medium in terms of rL, Y_L/X_ and biodiesel yield (Carota et al., 2020).

Although the FAME profiles of *R. toruloides* NRRL 1091 lipids changed significantly throughout the different phases of the fed-batch processes (Tables 2 and 3), they did not significantly impact on the estimated biodiesel properties. Regardless of the culture phase, however, relative concentrations of monounsaturated fatty acid were higher than 45%, a requisite giving excellent biodiesel properties such as fuel stability, combustion quality, exhaust emissions, and cold weather behavior (Pinzi et al., 2009). Similarly, total PUFA contents were well below the 30% of total lipids and thus fully compliant with the limits set out by the European Standard UNE-EN 14214 for the cetane number and iodine value, two parameters correlated with combustion quality and formation of combustion deposits, respectively (Ramos et al., 2009). Moreover, the FAME profiles of *R. toruloides* NRRL 1091 had a high resemblance to those of *Jatropha* (*Jatropha curcas* L.) oil, a conventionally used feedstock in biodiesel manufacturing.

## 5 Conclusions

Although carried out on a limited number of strains, this work shows that OPE can constitute a valuable basis of a lipid production medium for oleaginous yeasts. The thermal process yielding the D-limonene-free OPW, from which OPE derives, delivers a material amenable to storage at room temperature due to its low moisture content, thus ensuring its constant supply over time. Moreover, the obtention of OPE requires a simple aqueous extraction step, while other byproducts require time-consuming and expensive processing procedures such as acid and/or enzymatic hydrolysis. Although glycerol was generally ineffective in supporting biomass and lipid production for the strains tested, its formulation with the OPE-based medium in fed-batch processes allowed the achievement of promising lipid productivity with a net improvement compared to batch fermentation with the individual substrates. The present study shows that the feeding strategy of fed-batch cultivation had a strong influence on both biomass and lipid production by *R. toruloides* NRRL 1091. The continuous supply of N-containing OPE-glycerol mixtures at low dilution rates enabled the temporal extension of biomass and lipid production, avoiding at the same time the accumulation of OPE sugars in the medium. These findings confirm that the combination of glycerol with mixtures of easily assimilable carbohydrates, such as OPE, can boost lipid production by *R. toruloides* in agreement with other studies (Bommareddy et al., 2015; Rakicka et al., 2015; Galafassi et al., 2012). Although Ángel Siles López et al. (2010) outlined the potential of an orange waste-based biorefinery, only a few examples of integrated valorization of these residues are currently at hand (Lohrasbi et al., 2010; Balu et al., 2012). This study represents a further contribution to the development of OPW-based biorefineries.

## Declarations

### Author contribution statement

Eleonora Carota: Conceived and designed the experiments; Performed the experiments; Analyzed and interpreted the data; Wrote the paper.

Maurizio Petruccioli: Conceived and designed the experiments; Contributed reagents, materials, analysis tools or data; Wrote the paper.

Alessandro D’ Annibale: Analyzed and interpreted the data; Contributed reagents, materials, analysis tools or data; Wrote the paper.

Silvia Crognale: Performed the experiments; Analyzed and interpreted the data; Contributed reagents, materials, analysis tools or data; Wrote the paper.

### Funding statement

This work was supported by the 10.13039/501100003407Ministero dell'Istruzione, dell'Università e della Ricerca (MIUR) within the project "Piano Operativo Nazionale Biofeedstock" (grant no. ARS01_00985).

### Competing interest statement

The authors declare no conflict of interest.

### Additional information

No additional information is available for this paper.

## Appendix A Supplementary data

Figure_S1_Carota Figure S1. Diagram of the lipid production process including medium preparation from orange peel waste (OPW), fermentation trials, and subsequent chemical analyses conducted on biomass and culture supernatants. Figure_S1_Carota
