# Supplementary material for: Mixed glycerol and orange peel-based substrate for fed-batch microbial biodiesel production
Source: Heliyon. 2020 Sep 14;6(9):e04801. doi: 10.1016/j.heliyon.2020.e04801 (PMC7494470; doi:10.1016/j.heliyon.2020.e04801)
Supplement: Figure_S1_Carota — Figure S1. Diagram of the lipid production process including medium preparation from orange peel waste (OPW), fermentation trials, and subsequent chemical analyses conducted on biomass and culture supernatants. [file mmc1.pptx]

## Slide 1
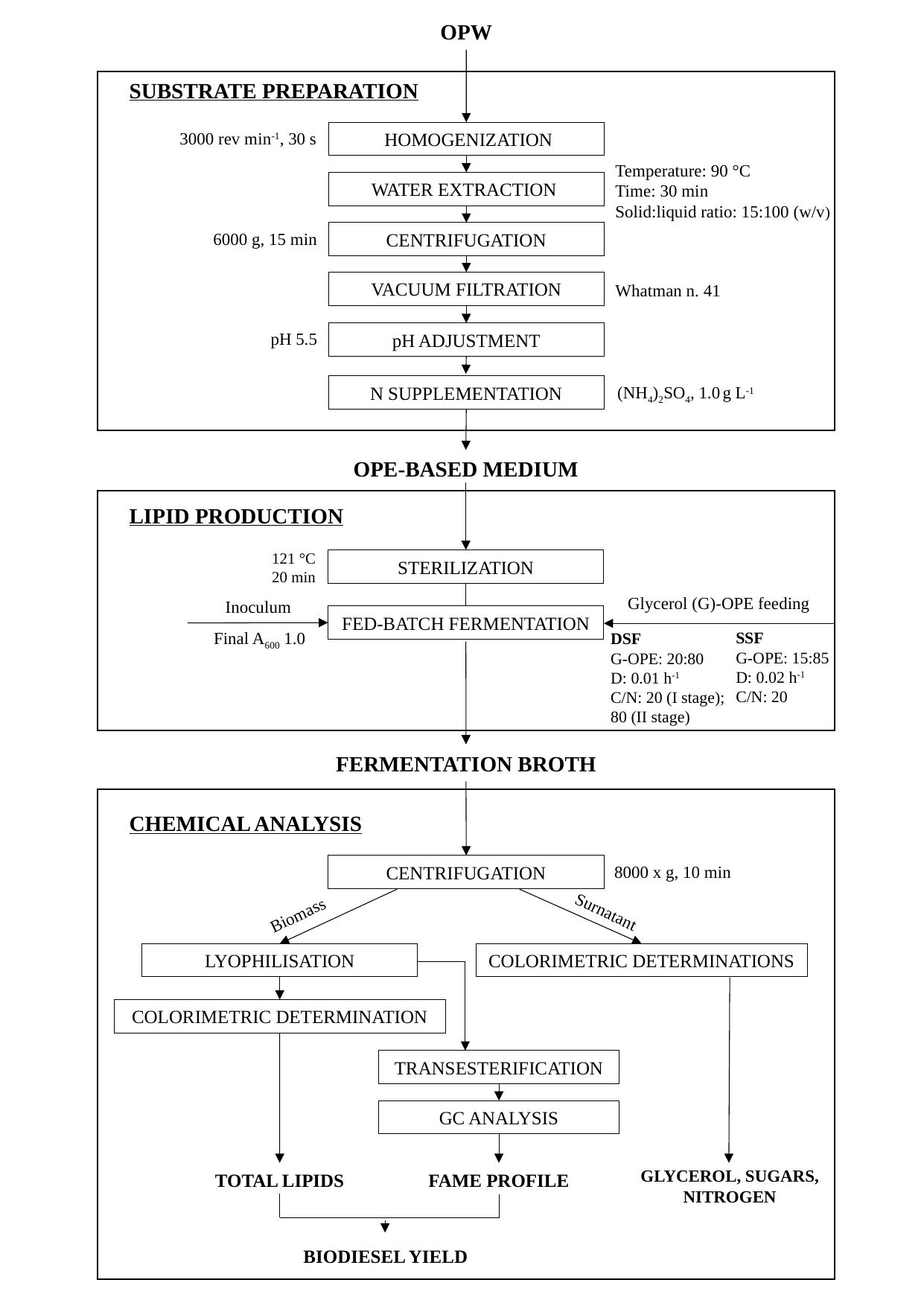

OPW
SUBSTRATE PREPARATION
 HOMOGENIZATION
3000 rev min-1, 30 s
Temperature: 90 °C
Time: 30 min
Solid:liquid ratio: 15:100 (w/v)
WATER EXTRACTION
6000 g, 15 min
CENTRIFUGATION
VACUUM FILTRATION
Whatman n. 41
pH 5.5
pH ADJUSTMENT
N SUPPLEMENTATION
(NH4)2SO4, 1.0 g L-1
OPE-BASED MEDIUM
LIPID PRODUCTION
121 °C
20 min
STERILIZATION
Glycerol (G)-OPE feeding
Inoculum
FED-BATCH FERMENTATION
Final A600 1.0
SSF
G-OPE: 15:85
D: 0.02 h-1
C/N: 20
DSF
G-OPE: 20:80
D: 0.01 h-1
C/N: 20 (I stage);
80 (II stage)
FERMENTATION BROTH
CHEMICAL ANALYSIS
CENTRIFUGATION
8000 x g, 10 min
Surnatant
Biomass
COLORIMETRIC DETERMINATIONS
LYOPHILISATION
COLORIMETRIC DETERMINATION
TRANSESTERIFICATION
GC ANALYSIS
GLYCEROL, SUGARS, NITROGEN
FAME PROFILE
TOTAL LIPIDS
BIODIESEL YIELD
